# Modelling the adhesion enhancement at the wheel–rail interface: the role of surface roughness and plastic deformation during rail sanding operation

**DOI:** 10.1007/s40534-025-00418-z

**Published:** 2026-02-14

**Authors:** Bin Zhang, Roger Lewis, Sadegh Nadimi

**Affiliations:** 1 School of Engineering, Newcastle University, Newcastle Upon Tyne, NE1 7RU UK; 2 Leonardo Centre for Tribology, Department of Mechanical Engineering, University of Sheffield, Sheffield, S13JD UK

**Keywords:** Wheel–rail adhesion, Particle breakage, Surface roughness, Plastic deformation, Finite element model, Cohesive interface elements

## Abstract

Efficient train operation relies on optimal traction at the wheel–rail interface, which can be compromised by factors such as water and/or contamination (e.g. leaf, sand, oil, and surface abrasion debris). This study introduces a finite element model to assess adhesion enhancement at the wheel–rail interface, with a focus on the impact of sand particles during the sanding process. Surface roughness is initially introduced to quantify its effect on adhesion, followed by the inclusion of rail plastic deformation. By integrating these two factors, the model provides a comprehensive framework for evaluating the complex mechanisms influencing adhesion at the wheel–rail interface, particularly in real-world train operations where surface conditions interact with contaminants such as sand fragments. This approach addresses the existing gap in understanding how rail surface condition and rail plastic deformation contribute to adhesion enhancement during the sanding process, offering new insights for optimising railway maintenance strategies.

## Introduction

Efficient and safe train operation relies on the adhesion coefficient in the wheel–rail interface. The level of adhesion coefficient in the wheel–rail interface significantly influences the performance and reliability of railway transportation systems [1, 2]. However, challenges such as wet weather, surface contamination, and variations in rail and wheel conditions can lead to reduced adhesion, posing significant operational risks [3–6]. In particular, low adhesion during traction operation leads to delays and general disruption due to a longer acceleration time. During braking operation, insufficient adhesion results in extended braking distances and may lead to signals passed at danger or collisions in extreme cases [7, 8]. When low adhesion is detected, rail sanding from an on-board device is utilised to increase the adhesion level during the wheel–rail contact [9, 10].

Rail grinding, employed as a maintenance measure to restore the worn rail surface [11–13], results in a very rough rail contact surface which can affect the adhesion coefficient and the influence of contamination. In order to evaluate the restored rail, roughness parameters have been adopted from metrology to quantify the surface after grinding. Mesaritis et al*.* [14] used a laboratory grinding process to investigate the effect of grinding parameters on the post-grinding roughness of the rail. Later, they utilised a full-scale testing facility to evaluate the performance of three rail grades after the grinding process [15]. Besides the quality control of the grinding process, Lundmark et al. [16] used surface roughness to quantify the tribological performance of the wheel/rail interface in the laboratory by using a two-disc rolling/sliding machine. They found that rougher wheel specimens resulted in lower wear compared to smoother specimens, particularly reducing the wear on the rail disc. Furthermore, Wang et al*.* [17] investigated the effect of surface roughness on friction and wear of the rail by adding different lubrications at the nanoscale. Their results indicate that reducing surface roughness, up to a critical point, can effectively reduce friction and wear under mixed lubrication conditions.

However, a roughness parameter by itself is not enough to monitor the adhesion level during train operation. Although the rail is made of steel, it suffers from cyclic loading that leads to permanent deformation, which also affects the adhesion level during the wheel–rail contact. Tomlinson et al*.* [18] used twin disc tests to simulate cyclic loading experienced by rail steel in service, from which a shear yield stress–plastic shear strain relationship can be generated for the tested sample. Later, Zhao and Li [19] used a three-dimensional (3D) transient finite element (FE) approach considering elasto-plasticity to explore the frictional rolling contact between the wheel and rail. They found the contact patch increased in size, shifted forward in the rolling direction, and changed from an ellipse into an asymmetric oval. Recently, Meyer et al*.* [20] developed a new methodology for finite element simulations of elasto-plastic rolling contact loading. It could provide higher accuracy for a given rolling length due to periodic boundary conditions.

Since the wear and plastic deformation of the rail occur simultaneously during train operation, it is not adequate to investigate the adhesion level at the wheel–rail interface by considering only one of these factors. As a result, Pletz et al. [21] developed a 3D finite element model of the full-scale test rig. Its result can be transferred to different two-dimensional models to calculate crack tip loading and cyclic deformations of rough surfaces. Following this, they introduced a quasistatic finite element model to calculate the plastic deformations of railway rails caused by rolling/sliding wheels [22]. By employing a 3D dynamic model, Vo et al. [23] found that a higher adhesion level enlarges slip regions and surface damage, while worn profiles alter the contact patch geometry and increase contact pressure. This contradicts the general understanding that wear causes the wheel and the rail to become more closely shaped (conformal), thus increasing the contact area and reducing the contact pressure. Recently, Spiryagin et al. [24] developed an algorithm to calculate the contact stresses with different surface roughness considering elastic and plastic deformations at the wheel–rail interface, emphasising the microscale mechanisms that alter adhesion conditions. Complementing this, Zhou et al. [25] demonstrated at the system level that nonlinear friction and wheel–rail adhesion are critical factors driving stick–slip vibrations in braking systems, thereby linking local adhesion variability to dynamic instabilities.

While research on the variation of adhesion during wheel–rail contact has seen advancements with the inclusion of additional parameters to replicate real-world conditions, there remains a scarcity of studies investigating the enhancement of adhesion resulting from the presence of a third body at the wheel–rail interface by incorporating the rail surface roughness and plastic deformation. This gap particularly relates to scenarios encountered during the sanding process in train operation. Studies [26–29] on the characterisation of sand particles during the sanding process have highlighted their critical role in adhesion enhancement, demonstrating how particle morphology, such as size and shape, influences the efficiency of adhesion improvement at the wheel–rail interface. Additionally, the FE model proposed by Zhang et al. [30] demonstrated a numerical framework for monitoring the adhesion coefficient at the wheel–rail interface. Therefore, following their study, this paper introduces an enhanced FE model to investigate the adhesion variation at the wheel–rail interface, triggered by sand fragments, surface roughness, and rail plasticity. The surface roughness is introduced to the model first to quantify its effect on adhesion enhancement. Then, the plastic deformation due to cyclic loading is assigned to the rail to investigate its influence on the existing adhesion enhancement contributed by surface roughness.

## Methodology

The adhesion enhancement triggered by sand particle breakage and fragments is quantified during the wheel–rail contact [30]. The whole operation is simulated through the finite element method (FEM) using Abaqus/Explicit package, and the breakage behaviour is achieved numerically using cohesive interface elements (CIEs).

### Breakage modelling

**Fig. 1:**
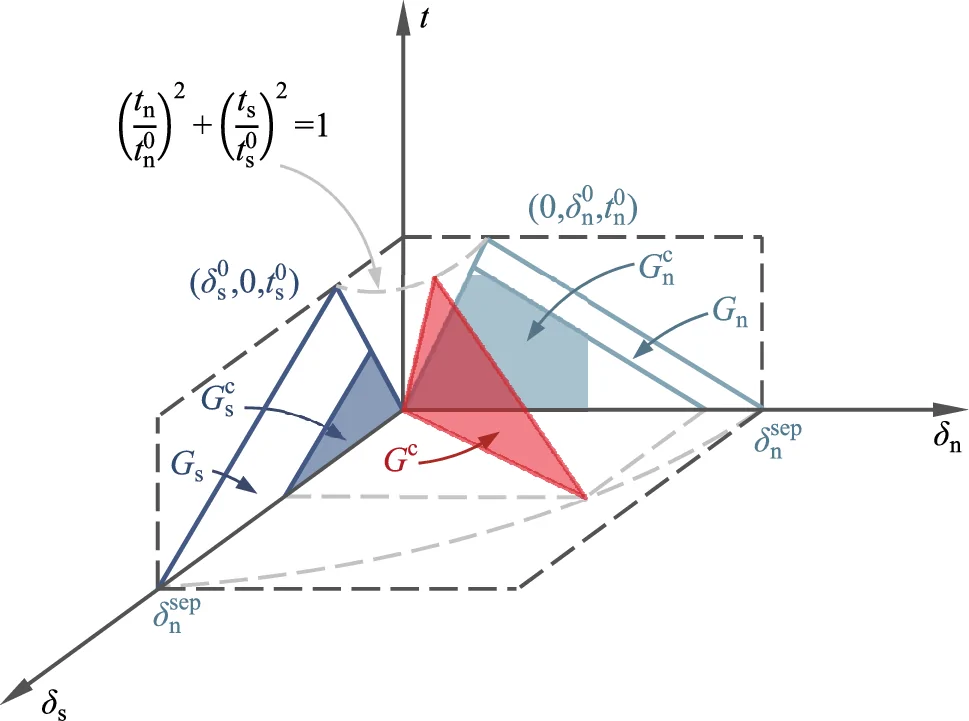
The quadratic nominal stress criterion and mixed-mode fracture criterion. ${\delta }_{\mathrm{s}}$ and ${\delta }_{\mathrm{n}}$ denote the nominal separation displacements in the normal and shear directions, respectively. Superscripts 0 and “sep” denote the values at damage initiation and complete failure, respectively

In FEM modelling, cohesive elements serve a valuable purpose in simulating adhesives, bonded interfaces, and fractures. For each application area, the constitutive response of these elements varies and mainly depends on certain assumptions regarding the related deformation and stress states. In this study, the traction–separation model has been adopted where the intermediate glue element is very thin and for practical purposes could be considered as zero thickness. The failure mechanism (see Fig. 1) of this model has two components: damage initiation criterion and damage evolution law.

#### Damage initiation

As the name indicates, the damage initiation marks the start of the degradation that occurs from a point in the material. The procedure of degradation begins when the stresses along the CIEs satisfy the selected damage initiation criteria. In this study, the quadratic nominal stress criterion is used and it assumes the damage will initiate when a quadratic interaction function including the nominal stress ratios reaches a value of one. This criterion can be illustrated as

1 $$\left( {\frac{{t_\mathrm{n} }}{{t_\mathrm{n}^{0} }}} \right)^{2} + \left( {\frac{{t_\mathrm{s} }}{{t_\mathrm{s}^{0} }}} \right)^{2} = 1,$$

where ${t}_{\mathrm{n}}$ and ${t}_{\mathrm{s}}$ represent the nominal stresses in normal and first shear directions, respectively, while ${t}_{\mathrm{n}}^{0}$ and ${t}_{\mathrm{s}}^{0}$ represent the maximum allowable nominal stresses in normal and first shear directions, respectively.

#### Damage evolution

Damage evolution can be characterised by energy dissipation during the damage process, which is also known as fracture energy. Considering the nature of two-dimensional modelling, only two modes of fracture have been involved, the normal mode (mode I) and the shear mode (mode II). The mixed-mode (mode I + mode II) fracture is adopted since it is the dominant failure behaviour of brittle materials compared to single-mode fracture. The Benzeggagh–Kenane (B–K) fracture criterion is utilised due to its efficiency in solving the critical fracture energies:

2 $$G^\mathrm{c} = G_\mathrm{n}^\mathrm{c} + \left( {G_\mathrm{s}^\mathrm{c} - G_\mathrm{n}^\mathrm{c} } \right)\left\{ {\frac{{G_\mathrm{s} }}{{G_\mathrm{n} + G_\mathrm{s} }}} \right\}^{\eta } ,$$

where ${G}^{c}$ is mixed-mode critical fracture energy. ${G}_\mathrm{n}^\mathrm{c}$ and ${G}_\mathrm{s}^\mathrm{c}$ are mode I and mode II fracture energies, respectively. ${\mathrm{G}}_{\mathrm{n}}$ and ${G}_{\mathrm{s}}$ are the fracture energy contributions in the normal and shear directions, respectively. η is a semi-empirical criterion exponent applied to delamination initiation and growth. Since there are limited studies on η of geo-materials, the parameter values of resin ranging from 2 (brittle) to 3 (ductile) are used in this study [31].

### Elastic and plastic behaviours

Elasticity indicates the capacity of a material to undergo deformation when subjected to a load and subsequently regain its initial shape upon load removal. For example, during the wheel–rail contact, the rail deforms elastically to a certain degree, allowing for better contact and adherence. The occurrence of excessive permanent deformation (plastic deformation) in rail materials is undesirable. Plastic deformation may promote wear and cause surface irregularities on the rail, negatively affecting adhesion, vehicle stability, ride comfort, and track durability. Therefore, it is critical to utilise a constitutive model that accurately represents how the material responds to loading. In this study, the rail is modelled with an elastic/plastic material description that uses a combination of isotropic and kinematic hardening.

The equivalent stress ${\sigma }_{\mathrm{v}}$ established by von Mises is employed to check the behaviour of a material in the principal plane stress case. Therefore, the yield stress ${\sigma }_{\mathrm{y}}$ can be expressed as follows to predict the plastic deformation:

3 $$\sigma_{{\mathrm{y}}} = \sigma_{{\mathrm{v}}} = \sqrt {\sigma_{1}^{2} - \sigma_{1} \sigma_{2} + \sigma_{2}^{2} } ,$$

where ${\sigma }_{1}$ and ${\sigma }_{2}$ are the two principal stresses. When ${\sigma }_{\mathrm{v}}\le {\sigma }_{\mathrm{y}}$, the material is dominated by the elastic deformation. As soon as the ${\sigma }_{\mathrm{v}}$ becomes bigger than ${\sigma }_{\mathrm{y}}$, the mechanical behaviour of material transmits from elastic to plastic.

The flow rule is another fundamental concept of plastic deformation that defines how the material behaves after the yielding point. It not only describes the relationship between stress and strain, but also specifies the direction and rate of plastic deformation.

**Fig. 2:**
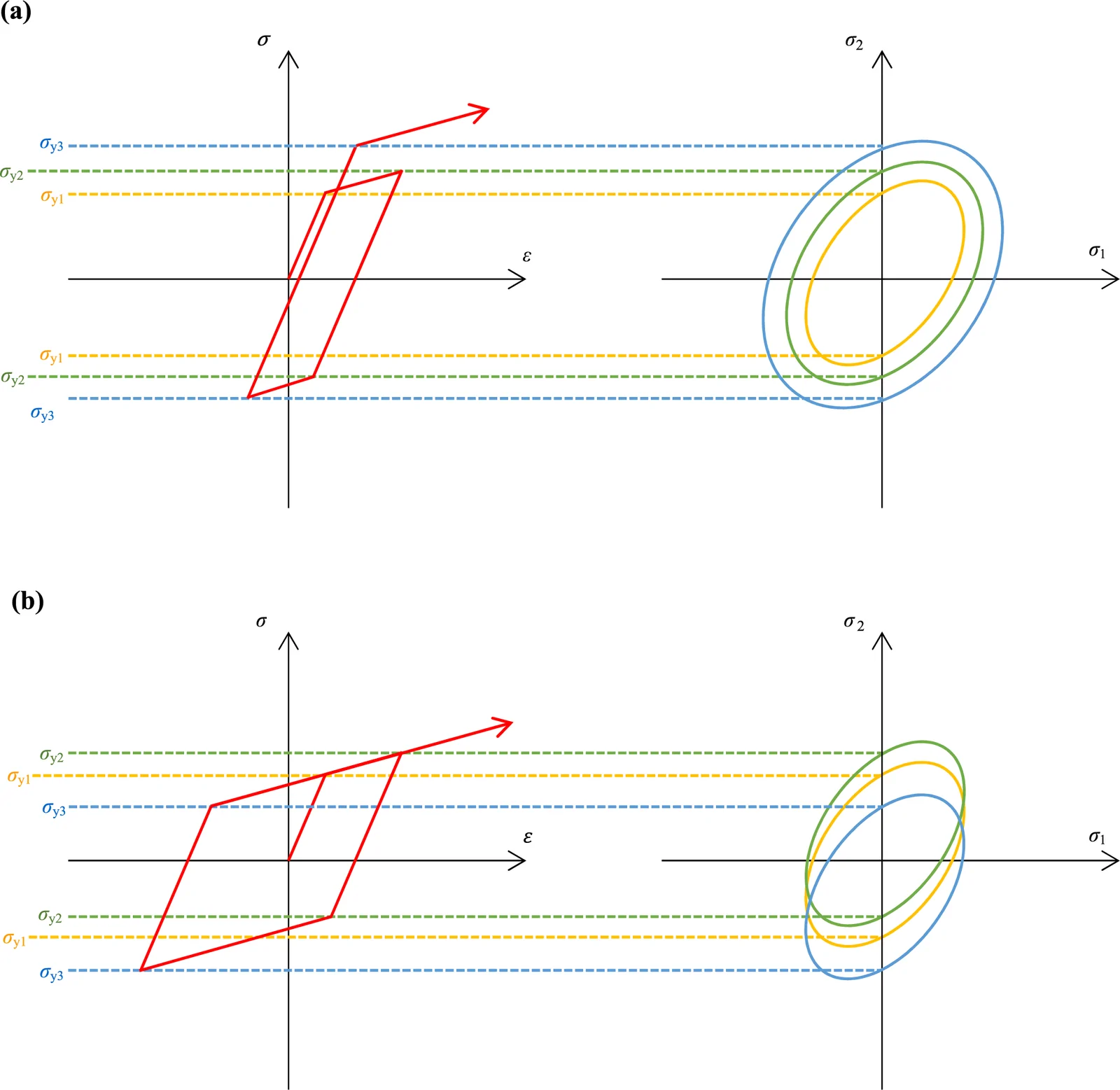
Two adopted flow rules: **a** isotropic hardening; **b** kinematic hardening. Here, σ is stress; ε is strain; ${\sigma }_{\mathrm{y}1}$, ${\sigma }_{\mathrm{y}2}$, and ${\sigma }_{\mathrm{y}3}$ are yield stresses corresponding to different shifts of the yield surface

Two flow rules are adopted in this study: (a) Isotropic hardening, where the yield stress ${\sigma }_{\mathrm{y}}$ increases in all directions with the accumulation of plastic strain. This strengthening effect occurs uniformly through the entire plastic deformation process. (b) Kinematic hardening, where the shape of the yield surface remains constant but its centre shifts. It is influenced by the accumulated plastic strain and past loading history. Figure 2 shows the changes in yield surface when the plastic deformation follows different flow rules.

### Roughness

Surface roughness constitutes a facet of surface texture, and it is characterised by the variances in the orientation of a real surface's normal vector from its idealised shape. In railway engineering, the roughness with a broad spectrum of wavelengths is present on the running surface of the rail. This rail roughness, in turn, triggers the generation of high-frequency wheel–rail contact forces, vibrations, and rolling noise. When the rails are worn beyond the specific allowable limits, their profiles must be restored to avoid undesired contact conditions in the wheel–rail interface. According to BS EN13231-3-2012 [32], the arithmetic mean surface roughness ${R}_{\mathrm{a}}$ of the rail should not exceed 10 µm along more than 16% of the measure length after grinding.

#### Data acquisition

**Fig. 3:**
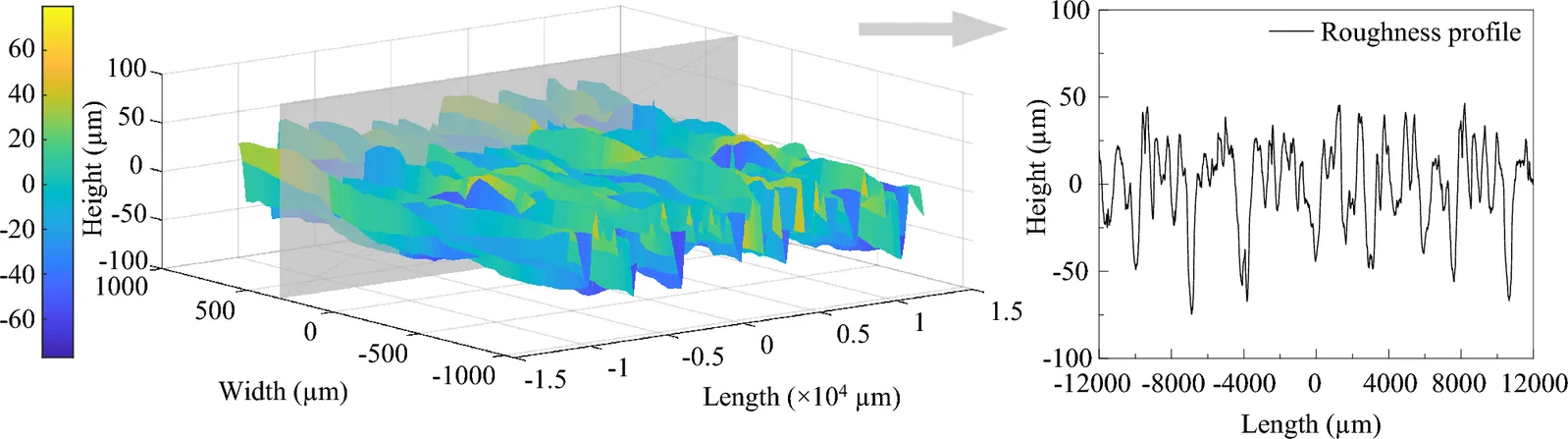
Representation of a high roughness profile (Case B)

The roughness of rail surface immediately after the grinding is measured following the procedure below [14]:

- Clean the surface using an acetone wipe.
- Apply a layer of Microset 101 Thixotropic replicating compound to the disc’s surfaces, add a piece of stiff backing paper, and wait 6 min for the replica to set.
- Use an Alicona InfiniteFocusSL 3D measurement system with a 5X magnification lens and a cut-off wavelength of 800 µm to create a digital representation of the surface of the replica.
- After digitisation, two sets of areas measuring 24 mm × 2 mm, obtained from real specimens, were provided by the industry partner. One set has medium roughness (*R*_a_ < 10 μm, labelled A), while the other has very high roughness (*R*_a_ ≈ 20 μm, labelled B). A typical surface profile is plotted in Fig. 3.

#### Data analysis

By using a lateral resolution of 4 µm, five hundred two-dimensional roughness profiles can be generated from each surface. In order to reduce computational complexity and make the analysis more manageable while preserving essential information, the original profiles have been performed a sub-sampling by a factor of 5 to reduce from five hundred to one hundred. The roughness descriptors listed in Appendix A are used to quantify each single roughness profile.

**Fig. 4:**
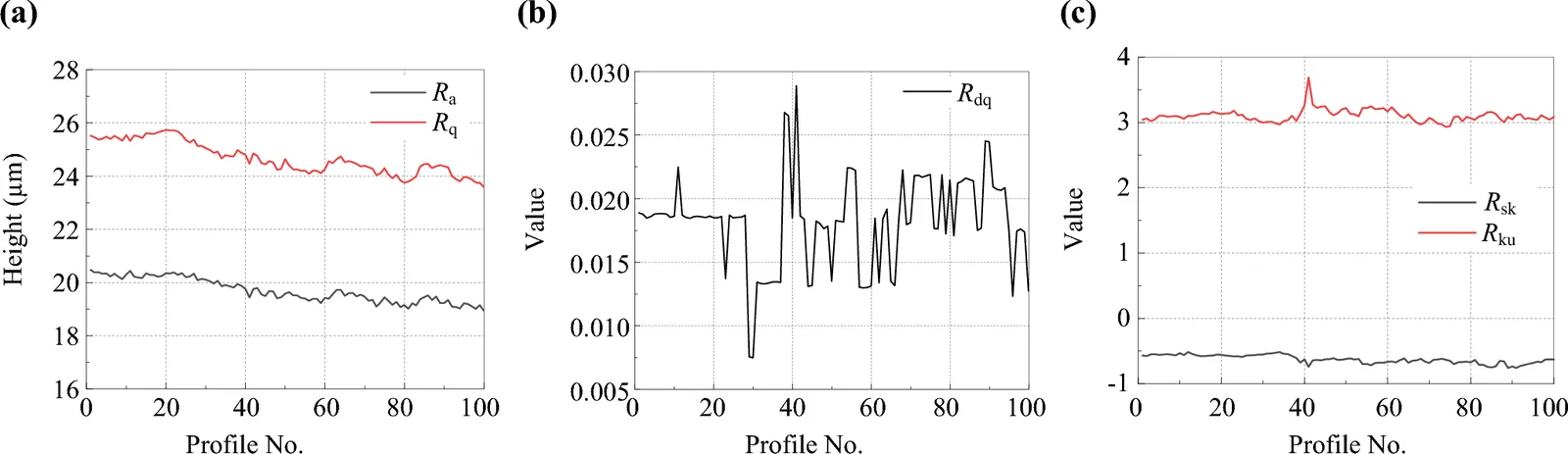
The roughness descriptors of 100 profiles from Case B: **a** ${R}_{\mathrm{a}}$ and ${R}_{\mathrm{q}}$; **b** ${R}_{\mathrm{dq}}$; **c** ${R}_{\mathrm{sk}}$ and ${R}_{\mathrm{ku}}$

Taking Case B as an example, the roughness variation trends are analysed in Fig. 4 using roughness descriptors defined in Appendix A. Clearly, all surface descriptors except ${R}_{\mathrm{dq}}$ exhibit stable behaviour: the ${R}_{\mathrm{a}}$ and ${R}_{\mathrm{q}}$ plots gradually decrease, while the ${R}_{\mathrm{sk}}$ and *R*_ku_ plots level off. As ${R}_{\mathrm{dq}}$ represents the general slope characteristics of the surface and serves as a distinguishing factor for surfaces with similar average roughness (${R}_{\mathrm{a}}$ and ${R}_{\mathrm{q}}$), a turning point analysis was performed on the ${R}_{\mathrm{dq}}$ data points. This analysis identified representative roughness profiles, resulting in the selection of 2 profiles from Case A and 35 profiles from Case B to characterise each surface area. The corresponding surface descriptors for these profiles are provided in Appendix B.

To ensure statistical representativeness, profiles with similar ${R}_{\mathrm{a}}$ were selected from Case B. This approach reduces the impact of specific profile variations on the simulation results and enhances the robustness and generalisability of the findings.

#### Profile regeneration

The next step is to generate the rail roughness in the numerical software. Each representative profile consists of over six thousand points. Therefore, a progressive data reduction has been conducted on each profile to reduce the geometrical complexity while still maintaining accuracy. The procedure is downsampling the data iteratively by selecting every nth point, where n starts from 2. For each downsampled dataset, ${R}_{\mathrm{a}}$ of the simplified profile has been calculated to compare with the original one. This downsampling process will continue until the error exceeds 1%, at which point the previous downsampled dataset will be adopted. Now, the representative profiles have been simplified while retaining the essential roughness characteristics within a certain error threshold.

From the previous study [30], a rail length of 110 mm is required to complete the traction and braking operations during the railway sanding process. Thus, each 24 mm representative profile has been duplicated to meet the requirement. A Python script has been developed to create rail parts reflecting the extended roughness profiles in Abaqus. The code first replicates each data point from the original 24 mm profile. Once the 24 mm profile is completed, the first data point from the original profile is reused to ensure continuity between the duplicated segments. To minimise discrepancies at the connection points, the roughness at the junction is kept lower than the ${R}_{\mathrm{a}}$ of the original profile. This approach ensures a smooth transition between the duplicated segments, maintaining the overall roughness consistency of the extended profile.

## Model set-up and materials

By utilising an existing two-dimensional wheel–rail model [30], the rail surface now has been amended by considering the roughness to simulate the interactions between sand fragments and uneven rail, which is a typical operating condition at the real wheel–rail interface. Additionally, in order to have a more realistic representation of complex material responses, a combined hardening material property has been assigned to the rail using the Abaqus/Explicit package to reproduce the effect from wear. This could provide a better simulation of the rail interaction under different loading conditions and further reveal the associated adhesion enhancement.

### Numerical model

**Fig. 5:**
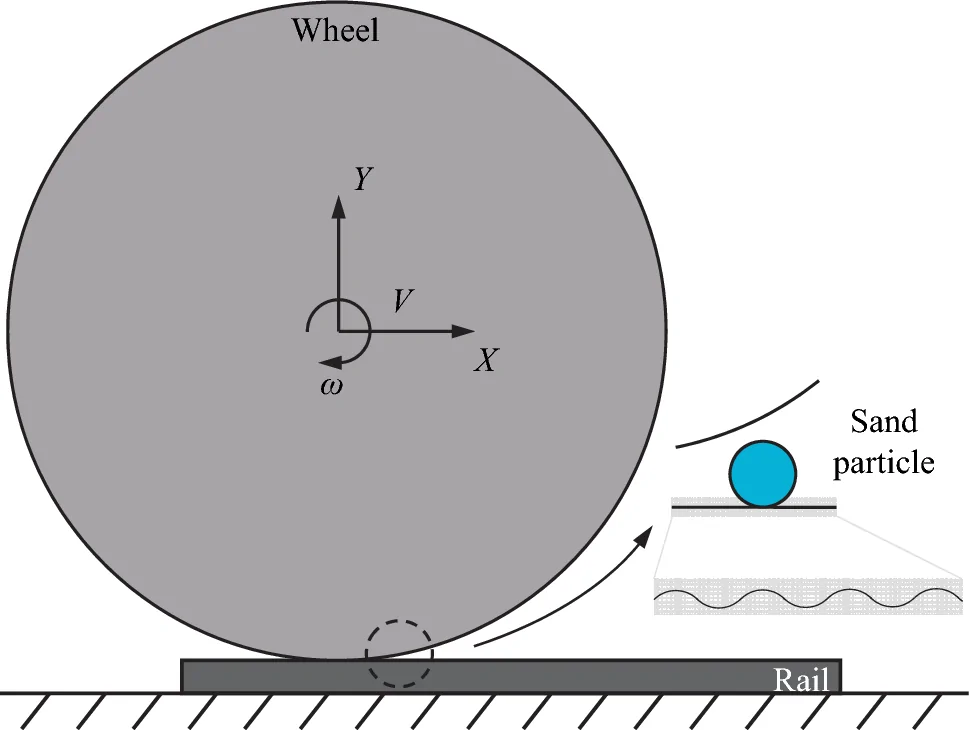
Schematic diagram of the wheel–rail model

As shown in Fig. 5, the wheel–rail model employed in this study comprises three components: (a) the wheel functions as a rigid body with displacement V in the X-direction. An angular velocity ω is switched on for traction operation and switched off for braking operation; (b) the rail is a deformable body that takes into account both elastic and plastic behaviours. It has been fixed at the bottom and the sides; (c) the sand particle operates as a deformable body equipped with cohesive interface elements to simulate fragmentation.

A concentrated normal force ${F}_{\mathrm{N}}$ of 60 kN is applied to the wheel. The tangential force ${F}_{\mathrm{T}}$ along the direction of motion is recorded throughout the entire simulation. As a result, the adhesion enhancement $\Delta \mu$ can be quantified using the equation below:

4 $$\Delta \mu = \frac{{F_{{\mathrm{T}}} }}{{F_{{\mathrm{N}}} }}.$$

In order to compare the adhesion enhancement from previous research [30], the sand particles used in this study range from 0.71 to 2 mm. Taking into account the average fragment size of 0.1 mm observed from experiments [33], a uniform element size of 0.1 mm is allocated to all sand particles. This element size effectively captures the realistic fragmentation process of sand particles at the wheel–rail interface. Furthermore, according to a mesh sensitivity analysis [34], the element size-to-mesh size ratio of the rail surface is set to 0.0005, which provides a balance between computational efficiency and accuracy (i.e. the execution time of simulations using 1 mm sand particle ranges from 2 to 4 h, depending on the complexity of the rough rail surface).

To achieve an accurate solution while maintaining a reasonable model size, non-uniform meshing has been applied to the rail. Since the ${R}_{\mathrm{a}}$ of the representative roughness profiles is in micrometres, finer mesh size is used at the wheel–rail contact within the roughness profile length (ca. 110 mm). It enables the capture of stress–strain behaviour of the rail surface influenced by the roughness on this micro-level. While the other side of the rail has been meshed using coarser elements. Beyond the roughness profile range, the rail is modelled as flat to expedite the simulation.

All deformable bodies are created using 3-node linear plane strain triangle (CPE3) elements, which can undergo local deformation based on the current nodal forces. In the case of sand particles, cohesive elements (COH2D4) are positioned at the interface of CPE3 elements. Since CIEs are of zero thickness, the overall mesh geometry remains unaltered, but every element is now connected by the CIEs. When the normal stress or shear stress at the CIEs reaches a predefined threshold, the CIEs initiate a process of vanishing, resulting in debonding between elements. The composition of sand particles used in this study is detailed in Table 1.

**Table 1:** Mesh composition of sand particles

Particle size (mm)	Element size (mm)	CPE3 elements
0.71	0.10	92
1.00	0.10	179
2.00	0.10	733

### Materials

Given that the wheel is a rigid body and does not require any material definition, the only two materials requiring definition are: (a) the sand particle, which employs a traction–separation model, and (b) the rail, which utilises a combined hardening model.

#### Sand particle

The material parameters of quartz sand have been carefully chosen to explore the adhesion enhancement triggered by sand fragmentation at the wheel–rail contact. Table 2 provides a summary of the material parameters utilised in this study, which encompass density (ρ), elastic modulus (E), Poisson’s ratio (ϑ), tensile strength $({N}_{\mathrm{max}})$, shear strength $({S}_{\mathrm{max}})$, CIEs’ stiffness $({k}_{\mathrm{n}}$ and ${k}_{\mathrm{s}})$, fracture energy $({G}_{\mathrm{n}}$ and ${G}_{\mathrm{s}})$, and material parameter (η). These parameters have been sourced from the previous study [30].

**Table 2:** Material parameters of sand particle

Parameter	Denotation	Value
*Solid elements (particle)*		
Density $(\mathrm{kg}/{\mathrm{m}}^{3})$	ρ	2500
Young’s Modulus $(\mathrm{GPa})$	E	63
Poisson’s ratio	ϑ	0.22
*Cohesive interface elements (CIEs)*		
Normal stiffness $(\mathrm{N}/{\mathrm{mm}}^{2})$	${k}_{\mathrm{n}}$	63,000
First shear stiffness $(\mathrm{N}/{\mathrm{mm}}^{2})$	${k}_{\mathrm{s}}$	31,500
Tensile strength $(\mathrm{MPa})$	${N}_{\mathrm{max}}$	25
First shear strength $(\mathrm{MPa})$	${S}_{\mathrm{max}}$	12
Mode I fracture energy $(\mathrm{N}/\mathrm{mm})$	${G}_{\mathrm{n}}$	0.1
Mode II fracture energy $(\mathrm{N}/\mathrm{mm})$	${G}_{\mathrm{s}}$	0.2
Material parameter	η	2
*Contact law*		
Particle-to-structure friction coefficient	μ	0.5

#### The rail

Since the rail exhibits both isotropic and kinematic hardening when undergoing external loads, the combined isotropic/kinematic model in Abaqus/Explicit package has been adopted to represent the realistic mechanical behaviours of the rail. It provides a more accurate approximation to the stress–strain relation and therefore a better prediction of the adhesion enhancement. The Young’s modulus and the Poisson’s ratio of the rail are 210 $\mathrm{GPa}$ and 0.3, respectively. The density of the rail is 7,800 $\mathrm{kg}/{\mathrm{m}}^{3}$.

##### Isotropic parameters

According to the work of Chaboche [35], the isotropic hardening can be described by a change in the yield stress ${\sigma }_{\mathrm{y}}$ shifting from its initial value ${\sigma }_{\mathrm{y}0}$, corresponding to the equivalent plastic strain ${\overline{\varepsilon }}_{\mathrm{pl}}$ in the following manner:

5 $$\sigma_\mathrm{y} = \sigma_\mathrm{y0} + Q_{\infty } \left( {1 - {\mathrm{e}}^{{ - b\overline{\varepsilon }_{{{\mathrm{pl}}}} }} } \right).$$

Here, according to Pletz et al. [21], the two material parameters ${Q}_{\infty }$ and b were established at −40 Mpa and 0.04, respectively. The initial yield stress ${\sigma }_{\mathrm{y}0}$ was measured as 320 MPa from the same study.

##### Kinematic parameters

The kinematic component of the hardening is represented through the yield function Y. In this function, the backstress tensor α is deducted from the stress tensor σ to simulate the kinematic hardening $Y(\sigma -\alpha )$. The α is calculated as the sum of each backstress ${\alpha }_{i}$ (*i* = 1, 2…). The incremental of ${\alpha }_{i}$ during plastic deformation is computed using the backstress rate ${\dot{\alpha }}_{i}$, which is derived from σ and the equivalent plastic strain rate ${\dot{\overline{\varepsilon }}}_{\mathrm{pl}}$ according to.

6 $${\dot{\alpha }}_{i}={C}_{i}\frac{1}{{\sigma }_{\mathrm{y}}}\left(\sigma -\alpha \right){\dot{\overline{\varepsilon }}}_{\mathrm{pl}}-{\gamma }_{i}{\alpha }_{i}{\dot{\overline{\varepsilon }}}_{\mathrm{pl}},$$

where the values of the material parameters ${C}_{i}$ and ${\gamma }_{i}$ are listed in Table 3. A final hardening curve is generated by combining the listed six backstress curves to provide a better capture of the shape of the plastic behaviour.

**Table 3:** Backstress parameters of the railhead part [21]

i	${C}_{i}\, (\mathrm{GPa})$	${\gamma }_{i}$
1	350	5000
2	80	1000
3	15	150
4	10	50
5	8	20
6	0.19	0.01

By incorporating these two parameters, the model can simulate the hardened layer that forms on the rail surface due to cyclic loading. However, since the primary objective of this study is to investigate the effect of surface roughness on adhesion at the wheel–rail interface, the analysis focuses exclusively on the critical case of the first loading cycle after grinding, where the yield strength of the rail is lower than that of the hardened rail. Nevertheless, this setup establishes the numerical foundation for future investigations involving cyclic loading.

### Parametric study

Three particles with diameters of 0.71, 1, and 2 mm are used to investigate adhesion enhancement on a selected rough surface, with results compared to those on a flat surface previously reported by Zhang et al. [30]. Both traction and braking operations are simulated to replicate the actual sanding process. For this analysis, only the elastic deformation of the rail is considered, ensuring a focused examination of the particle size effect due to surface roughness.

#### Traction operation

**Fig. 6:**
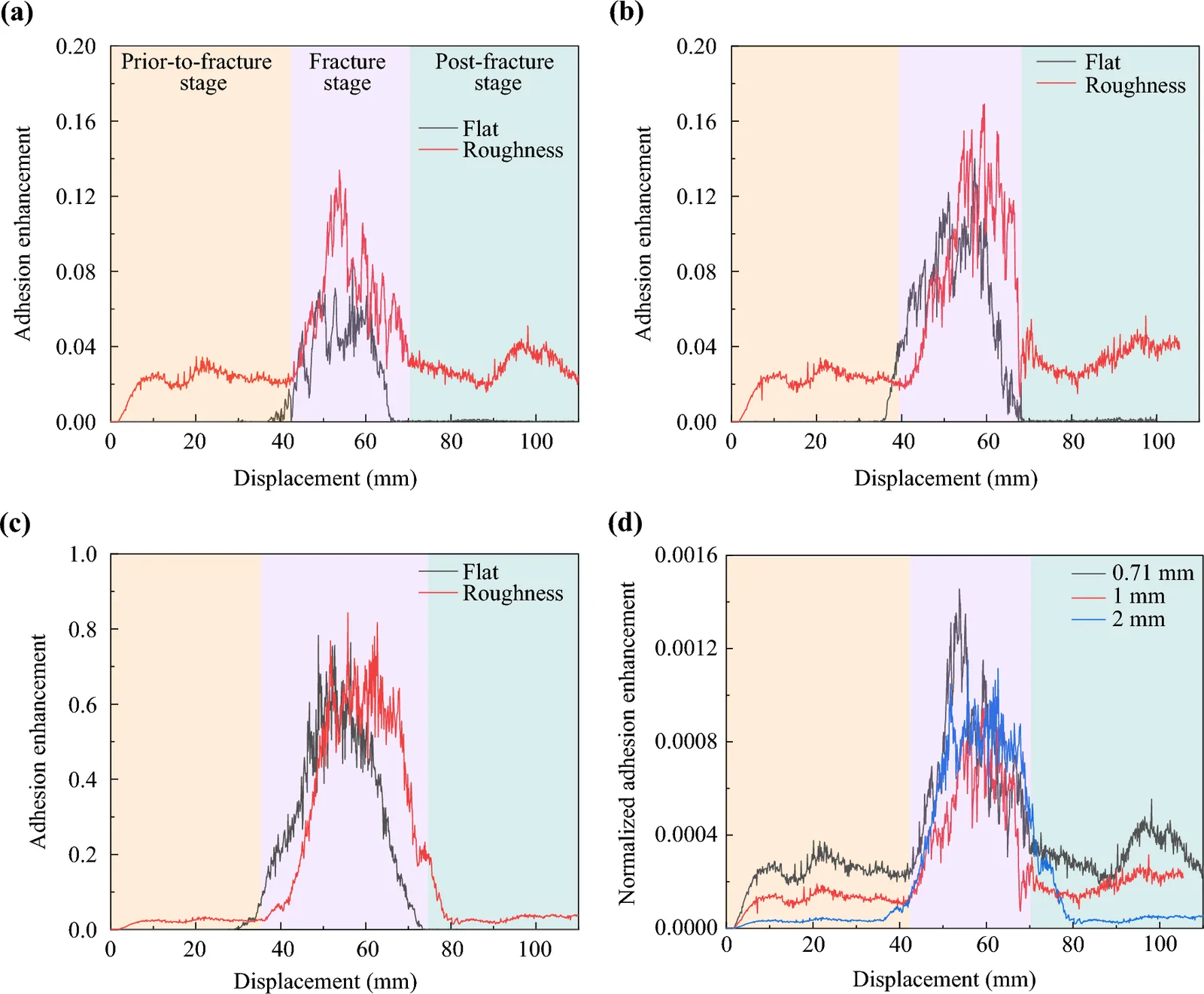
Size effect during traction operation: **a** 0.71 mm; **b** 1 mm; **c** 2 mm; **d** normalisation

The change of adhesion during traction operation is plotted in Fig. 6. Using Fig. 6a as an example, displacement smaller than 45 (40 for the flat surface) is defined as the prior-to-fracture stage and bigger than 70 (65 for the flat surface) is defined as the post-fracture stage. For these two stages, the wheel is only interacting with the rail. From the displacement of 45 to 70 (40 to 65 for the flat surface), the wheel is rolling on top of the sand fragments, which is considered as the fracture stage. By comparing the two profiles using the same particle size, although the roughness case takes a longer time to achieve the fracture stage due to the uneven surface contact, the durations of the fracture stage are the same for both flat and rough surfaces. However, when comparing fracture stages for simulations using different particle sizes, the duration becomes longer as a result of particle size increment.

It is noteworthy that the adhesion enhancement on rough surfaces during prior-to-fracture and post-fracture stages is identical for all simulations as no particle is involved. However, the peak value during the fracture stage varies from particle size to particle size. In order to reveal the effect of particle size on adhesion enhancement, the data are normalised based on their respective fragment counts, as shown in Fig. 6d. During the prior-to-fracture and post-fracture stages, since no fragments are involved, the plots from small particles show higher values than the large particles due to the normalisation. However, the participation of fragments during the fracture stage harmonises this difference, as the plots from different particle sizes converge. This indicates the new surface area generated in the contact due to fragmentation is similar after the normalisation.

Additionally, similar variation trends have been observed in the results from simulations using different particle sizes on the same rough surface during the fracture stage. While the magnitudes vary due to the different amounts of fragments involved, the results converge after normalisation. These similar trends demonstrate the model’s repeatability and reliability.

#### Braking operation

**Fig. 7:**
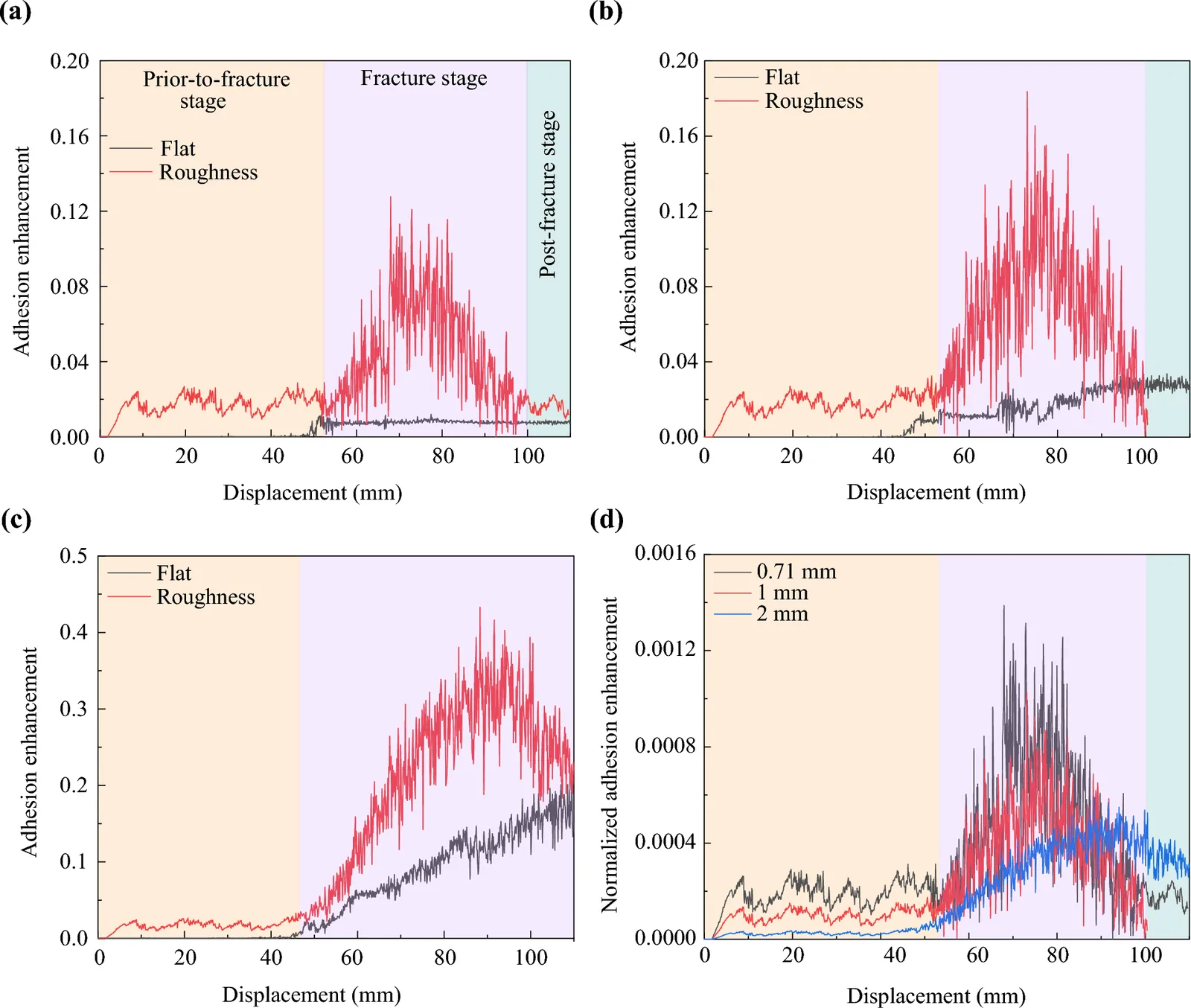
Size effect during braking operation: **a** 0.71 mm; **b** 1 mm; **c** 2 mm; **d** normalisation

When the train is approaching a station or designated stop, in the worst-case scenario, the wheel is only sliding with no rotation to decelerate the train from its current speed to a complete halt. Figure 7 shows the observed adhesion enhancements within this operation. Depending on the participation of sand fragments, the braking operation can also be separated into three stages: the prior-to-fracture stage, the fracture stage, and the post-fracture stage. The adhesion enhancement during the prior-to-fracture stage is comparable for particles of different sizes. Compared to traction operation, for rough surface, the duration of the fracture stage increases tremendously for bigger particles during the braking operation, and therefore the post-fracture stage is not well observed due to the predefined run-length of the model. However, based on the plots from small particles and the stage definition, the post-fracture stage for bigger particles will be similar to its prior-to-fracture stage and only require a longer time to be achieved. This observation introduces a significant difference from the result using the flat surface where the adhesion enhancement increases sharply to its peak value during the fracture stage and then carries the value over into the post-fracture stage till the end of the simulation.

Similarly, a normalisation is conducted on the data and is plotted in Fig. 7d. The initial difference in plots during the prior-to-fracture stage has been discussed in the previous section. Although the fracture stage still lasts longer for bigger particles, its peak value now is comparable to results from small particles.

#### Fragment distribution

**Fig. 8:**
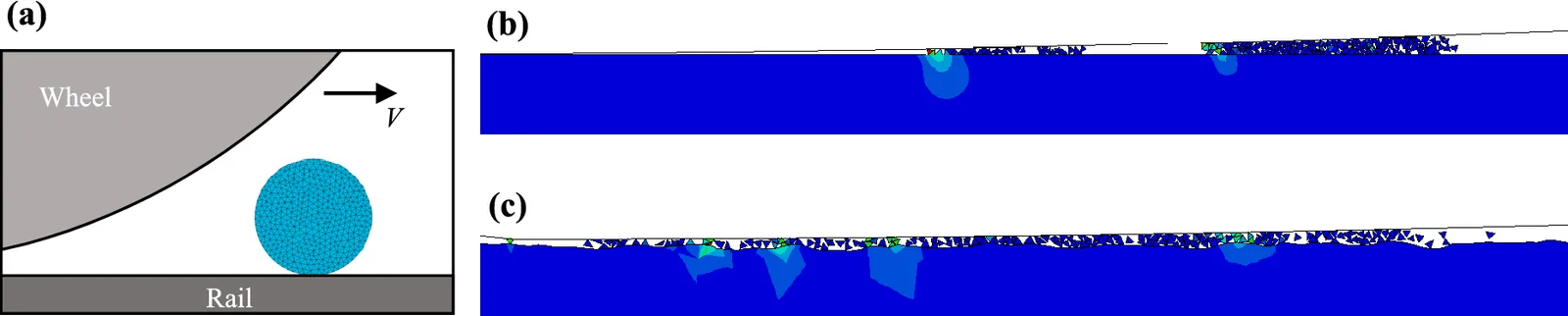
Fragment distribution at the fracture stage: **a** the configuration of 1 mm sand particle during braking operation; **b** on the flat surface; **c** on the rough surface

According to the observations during the breakage of sand particles, the fragment distribution of a rough surface during traction operation is similar to a flat surface, which has been investigated in a previous publication by Zhang et al. [30]. However, during the braking operation, the adhesion enhancement behaves differently on flat and rough surfaces. This requires a further investigation of the fragment distribution during the wheel–rail contact to reveal the underlying facts. For example, the observations of 1 mm sand particle at the fracture stage are plotted in Fig. 8. In the flat case, the sand fragments have been held and pushed away by the sliding wheel till the end of the simulation. Thus, the number of fragments entering the wheel–rail interface starts to increase till a certain value and then this value will be maintained. This explains the reason why adhesion enhancement increases sharply at the beginning and then becomes stable later on. In contrast, the interlocks between the fragments and the uneven surface generate reaction forces which causes the deformation of the rail. Hence, instead of being pushed away, this deformation enables the sand fragments to be passed by the rolling wheel. Consequently, the plots in Fig. 7 illustrating initial increases in adhesion enhancement, followed by a subsequent decline, have been generated.

In summary, the magnitude of adhesion enhancement in the rough case is depending on the number of fragments entering the wheel–rail interface rather than the particle size. This aligns with the earlier findings using a flat rail [30]. Furthermore, the rough surface permits the wheel to roll over the fragments during braking operation, a feature distinct from the flat surface.

## Results and discussion

A total of 37 representative roughness profiles from cases A and B are analysed to examine the effect of surface roughness and rail plastic deformation on adhesion enhancement at the wheel–rail interface during traction and braking operations. To ensure consistency and comparability, particles with a 1 mm diameter are used hereafter.

### Roughness effect

To isolate and quantify the effect of surface roughness on adhesion, only the elastic deformation of the rail is considered in this section, allowing for a better understanding of how roughness alone contributes to the overall adhesion enhancement at the wheel–rail interface.

#### Traction operation

**Fig. 9:**
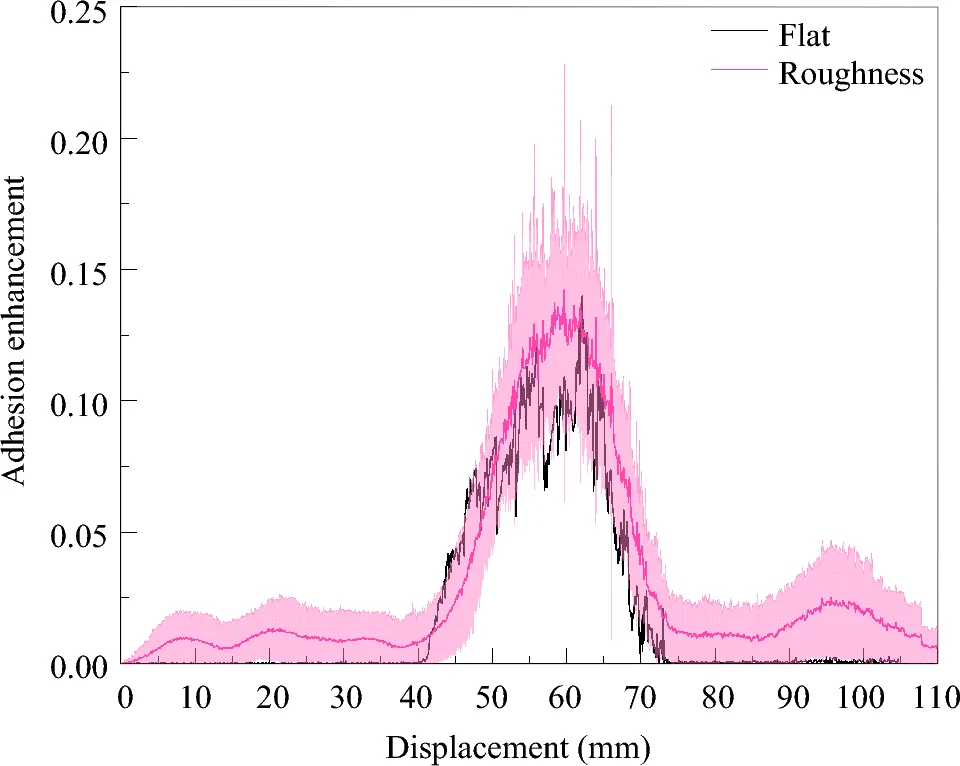
Roughness effect during traction operation

The results from 37 simulations using rough surfaces under traction operation are plotted in Fig. 9 to compare with the flat surface. The red solid line indicates the average value of the 37 simulations, and the pink shade demonstrates its standard deviation. The black line shows the result of adhesion enhancement from the flat surface.

By comparing the two solid lines, the observation from the previous section using the single roughness profile has been confirmed. Furthermore, the smooth portion of the red line during the prior-to-fracture stage indicates a stable improvement of adhesion due to rough surfaces. It begins to rise shortly when the fracture stage occurs. After a sharp curve, the adhesion begins to decrease in a pattern similar to how it had previously increased. It ends with an adhesion enhancement level comparable to that of the prior-to-fracture stage. This variation trend is identical to the black line from the flat surface. The difference in peak values between the two solid lines can be correlated with the initial improvement due to roughness.

#### Braking operation

**Fig. 10:**
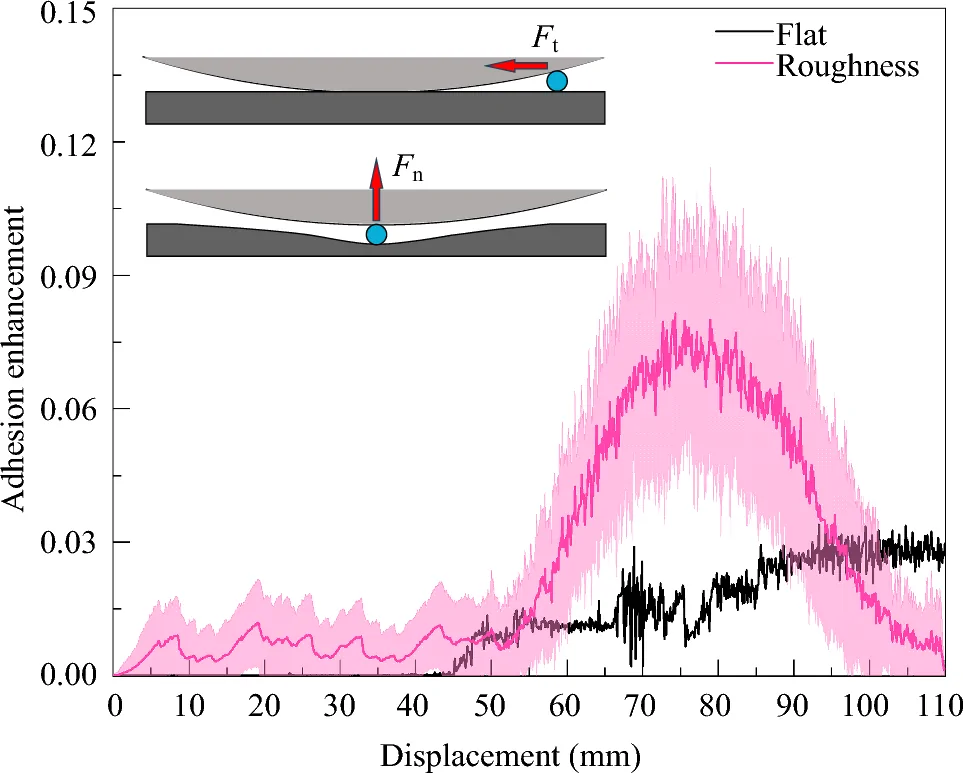
Roughness effect during braking operation

The simulations are repeated for the braking operation. Similarly, as illustrated in Fig. 10, the average value of adhesion enhancements from 37 simulations is represented by the red solid line, with the pink-shaded area indicating its standard deviation. The adhesion enhancement obtained from the flat surface is depicted by the black line. The hill-shaped plot of average value proves the observation of fragment distribution in the previous section that the mechanical interaction of sand fragments gets changed when switching from the flat surface to the rough surface. Rather than being repelled, the fragments can pass through the wheel–rail interface using the gaps between the wheel and the uneven rail.

During the prior-to-fracture stage, the plot of the red line indicates the adhesion can be improved during braking operation as well. Although its value is comparable to the enhancement during traction operation, the zigzag shape shows the unstable reaction between the sliding wheel and uneven rail. This phenomenon persists throughout the entire operation, even when sand fragments are present, leading to significant noise in the hill-shaped plot during the fracture stage. Due to the horizontal force from the sliding wheel, it takes longer to complete the fracture stage compared to traction operation. The peak value generated from rough surfaces is about three times higher than the one from the flat surface. This is owing to the total normal force has been reduced by the reaction force ${F}_{\mathrm{n}}$ generated from the fragments entering the wheel–rail interface, leading to a higher adhesion level compared to the result from the flat surface where no normal force reduction exists, as illustrated by the inset of Fig. 10. However, this peak value is still lower than the one from the rough surface during traction operation as the rolling wheel contributes to a higher total tangential force.

#### Roughness parameter

Based on the earlier discussions, it is evident that rough surface significantly influences the adhesion in both traction and braking operations. Hence, the widely used descriptor, ${R}_{\mathrm{a}}$, is employed here to examine the contribution of roughness to adhesion enhancement. It is a general indicator of surface finish by specifying the average height deviations. Since adhesion enhancement during the post-fracture stage in both traction and braking operations behaves similarly to the prior-to-fracture stage, the only first two stages (i.e. prior-to-fracture stage and fracture stage) are considered here.

**Fig. 11:**
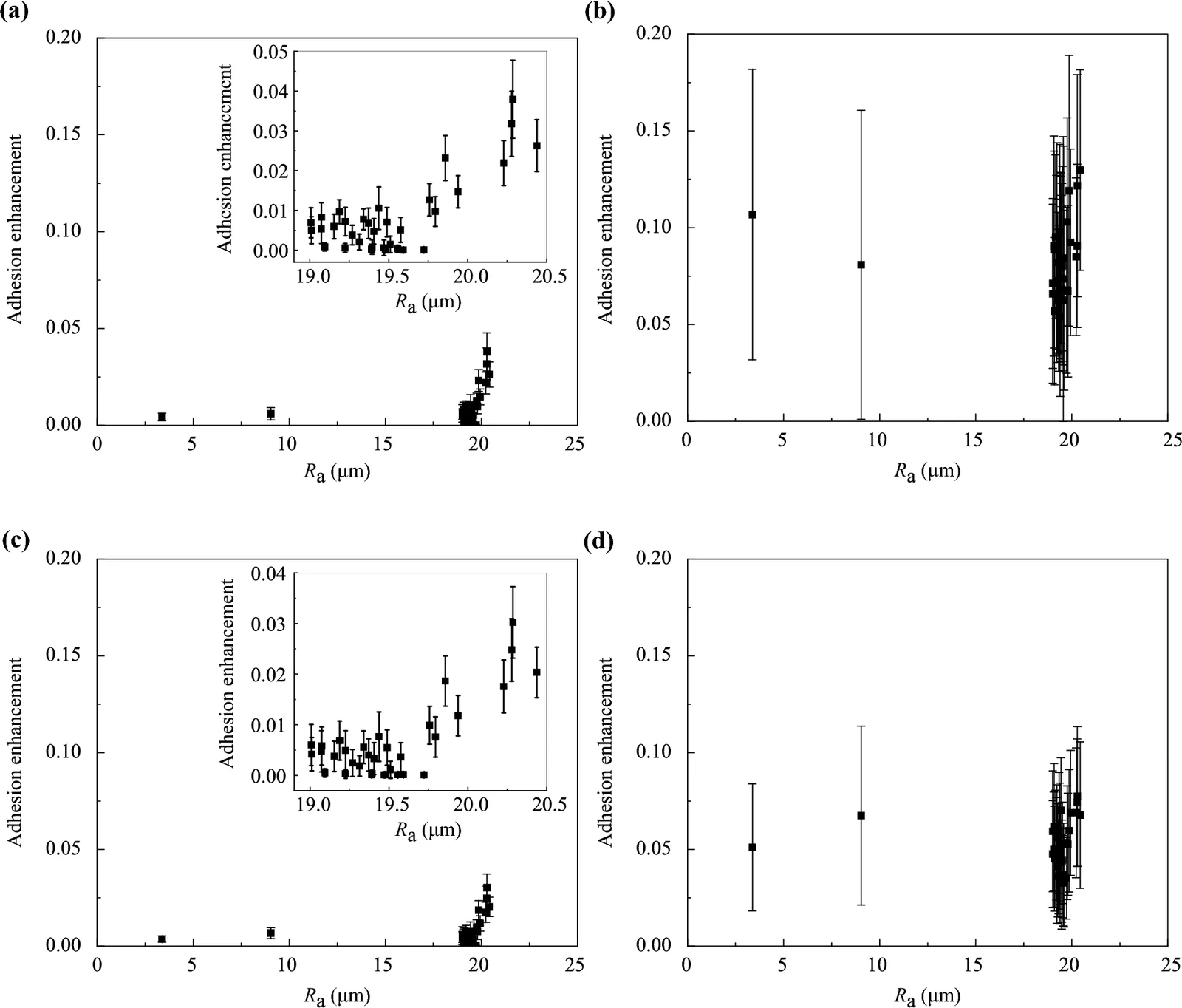
Adhesion enhancements influenced by ${R}_\mathrm{a}$: **a** prior-to-fracture stage; **b** fracture stage during traction operation; **c** prior-to-fracture stage; **d** fracture stage during braking operation

As shown in Fig. 11, the black square indicates the average value of adhesion enhancements during the stage affected by different ${R}_\mathrm{a}$ and the flat end bars represent its standard deviation. By comparing Fig. 11a and c, although the adhesion enhancement does not show a significant rise, it is clear that the standard deviation increases with the rise of ${R}_\mathrm{a}$, suggesting that a rougher surface tends to induce greater fluctuations in adhesion. Additionally, the values exhibit comparability between traction operation and braking operation during the prior-to-fracture stage. It signifies the adhesion level is not affected by the mode of operation when sand fragments are not involved. However, this relationship is changed when the fracture stage begins, as illustrated in Fig. 11b and d. While the adhesion enhancement is stable during both operations, the average value and standard deviation of adhesion enhancement are bigger during traction operation than the ones during braking operation. This indicates that adhesion experiences a tremendous fluctuation at the fracture stage during traction operation. In addition, the values of adhesion enhancement are levelled up during the fracture stage compared to the prior-to-fracture stage, as the same amount of fragments were generated during particle breakage. This observation suggests a bigger ${R}_\mathrm{a}$ contributes to a higher adhesion enhancement during the prior-to-fracture stage where no sand particles are involved, and its influence becomes less pronounced compared to the effect of sand fragments during the fracture stage.

### Plasticity effect

The interlocking between sand fragments and the rough surface generates forces that deform the rail profile, allowing more fragments to enter the wheel–rail interface. When only elastic deformation is considered, the recovery of rail deformation introduces reaction forces that increase the adhesion level. However, in practice, plastic deformation, such as wear, typically results in a lower adhesion level. Therefore, it is crucial to use a numerical model that accounts for plastic deformation to further investigate the effects of roughness. After defining the plastic parameters of the rail, the previous model now can be used to further investigate the adhesion level in a more realistic way.

#### Traction operation

**Fig. 12:**
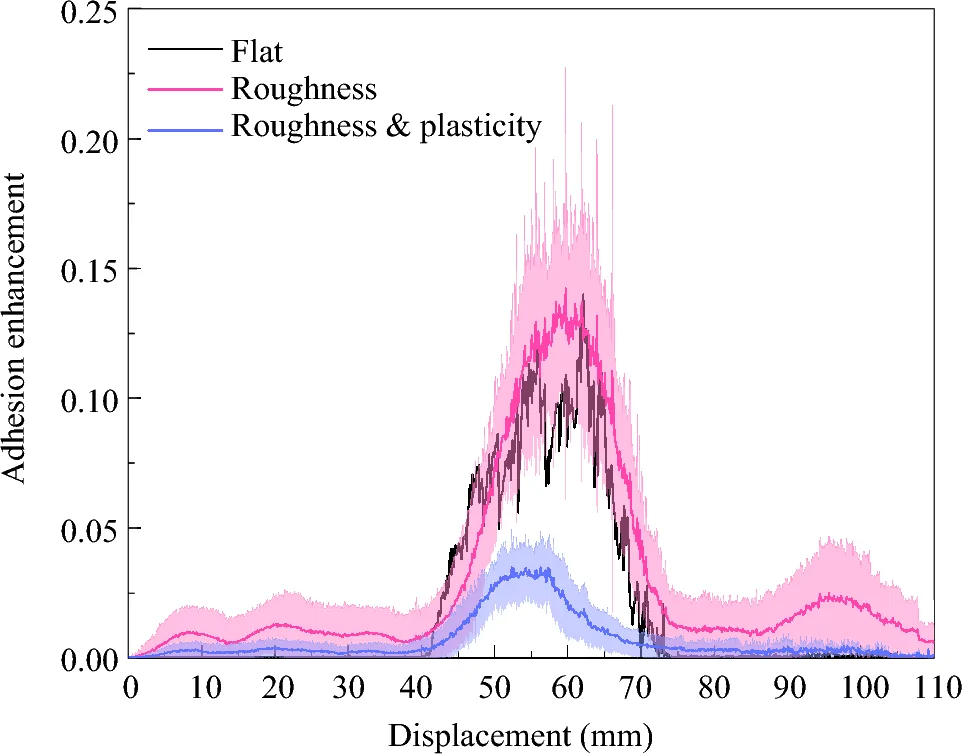
Plasticity effect during traction operation

As shown in Fig. 12, the blue shaded area represents the standard deviation of results considering both elastic and plastic deformation, while their mean value is depicted as a continuous blue line. Although the trends of variation are similar, the values from roughness cases that account for plasticity are significantly smaller than those considering only elasticity, with a reduction of approximately 60%. This causes the values to be even smaller than those observed in the flat case. The reduction is attributed to the permanent deformation in the rail caused by its plastic properties, which diminishes the reaction force generated by the recovery of elastic deformation, thereby reducing the adhesion enhancement. Moreover, compared to the red plots, the width of the blue shaded area becomes narrower, and the noise in the blue solid line appears smoother, with a plateau forming at the peak during the fracture stage. All these observations suggest that the enhanced model, which considers plasticity, results in a more stable adhesion level.

#### Braking operation

**Fig. 13:**
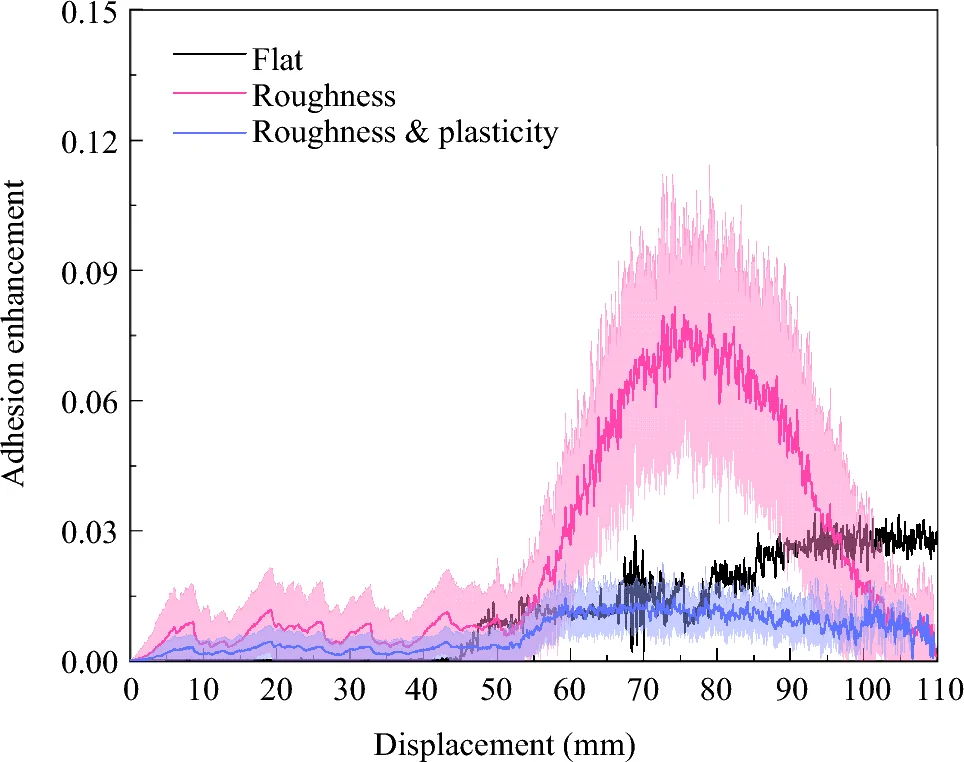
Plasticity effect during braking operation

The plasticity also affects the adhesion level during braking operation, as shown in Fig. 13. Due to the sliding wheel during braking operation, the plots from all cases exhibit a persistent vibration throughout the entire simulation. However, their variation trends behave differently. The great divergence starts during the fracture stage. Although all the plots begin to increase at the same time, the slope and peak values vary between cases. For the flat case, the plot starts with a gentle slope and transitions into a plateau that continues until the end. For the roughness case, which only considers elasticity, the plot rises rapidly and falls sharply, reaching a higher peak value compared to the flat case. When plasticity is included in the rough case, the plot ascends with a gentle slope, transitions into a prolonged plateau at half the peak value of the flat case, and then descends with an even more gradual slope.

Overall, this enhanced model reveals the significant impact of plastic deformation on adhesion enhancement during both traction and braking operations. While roughness without plasticity produces higher peak adhesion, it exhibits greater variability and instability, as indicated by the wider shaded regions and sharper transitions. In contrast, the inclusion of plastic deformation in the model leads to a more stable adhesion response, with smoother transitions and prolonged plateaus at lower adhesion levels. However, the reduction in peak adhesion due to plasticity highlights the importance of routine rail grinding to mitigate the effects of wear and maintain the desired surface roughness for optimal adhesion during sanding. These findings emphasize the need for numerical models that incorporate both elastic and plastic behaviours to accurately predict adhesion performance and the importance of consistent rail maintenance to ensure safe and efficient traction and braking in railway operations.

## Conclusions

By considering the surface roughness and plastic deformation of the rail, this study developed a finite element model to investigate adhesion enhancement at the wheel–rail interface, triggered by fragments from sand particles. The effects of roughness and plastic deformation on adhesion were observed and analysed, leading to the following key conclusions:

- Adhesion enhancement triggered by sand particles is size-independent and can be correlated with the number of generated fragments, regardless of the rail surface finish.
- Surface roughness significantly influences adhesion levels during the pre-fracture stage but becomes less impactful during the fracture stage, where the presence of fragments dominates adhesion behaviour.
- Plastic deformation in the rail reduces the reaction forces from fragments during wheel–rail contact, ultimately leading to a lower overall adhesion level.

The methodology employed in this study offers valuable new insights into adhesion behaviour at the wheel–rail interface by incorporating surface roughness and plastic deformation parameters. This approach provides a more realistic simulation environment, paving the way for future studies to refine the understanding of wheel–rail interactions and adhesion mechanisms.

## Appendix A

See Table 4.

**Table 4:** Supported rail roughness descriptors

Roughness descriptor	Formula	Range	Comments
Arithmetical mean height $({R}_{\mathrm{a}})$	${R}_{\mathrm{a}}=\frac{1}{{\ell}}{\int }_{0}^{{\ell}}\left|Z\left(x\right)\right|\mathrm{d}x$	[0,+∞)	Units of length
Root mean square height $({R}_{\mathrm{q}})$	${R}_{\mathrm{q}}=\sqrt{\frac{1}{{\ell}}{\int }_{0}^{{\ell}}{Z}^{2}\left(x\right)}\mathrm{d}x$	[0,+∞)	Units of length
Root mean square gradient ${(R}_{\mathrm{dq}})$	${R}_{\mathrm{dq}}=\sqrt{\frac{1}{{\ell}}{\int }_{0}^{{\ell}}{\left[\frac{\mathrm{d}}{\mathrm{d}x}Z\left(x\right)\right]}^{2}}\mathrm{d}x$	[0,+∞)	Unitless
Skewness $({R}_{\mathrm{sk}})$	${R}_{\mathrm{sk}}=\frac{1}{{R}_{\mathrm{q}}^{3}}\left[\frac{1}{{\ell}}{\int }_{0}^{{\ell}}{Z}^{3}\left(x\right)\mathrm{d}x\right]$	$(-\infty ,+\infty )$	Unitless
Kurtosis $({R}_{\mathrm{ku}})$	${R}_{\mathrm{ku}}=\frac{1}{{R}_{\mathrm{q}}^{4}}\left[\frac{1}{{\ell}}{\int }_{0}^{{\ell}}{Z}^{4}\left(x\right)\mathrm{d}x\right]$	[0,+∞)	Unitless

Note: x is the position along the rail profile, ℓ is evaluation length of the rail profile, and *Z*(*x*) is the vertical deviation of the measured rail surface from the mean reference line at position *x*

## Appendix B

See Table 5.

**Table 5:** List of representative rail head profiles

Profile ID	*R*_a_ (μm)	*R*_q_ (μm)	*R*_dq_	*R*_sk_	*R*_ku_
A-1	3.384	4.314	0.004	−0.119	3.662
A-2	9.037	12.092	0.011	−1.103	−1.103
B-1	20.228	25.399	0.019	−0.554	3.107
B-2	20.438	25.328	0.022	−0.563	3.058
B-3	20.279	25.595	0.019	−0.571	3.128
B-4	20.286	25.682	0.014	−0.584	3.181
B-5	20.229	25.267	0.019	−0.568	3.054
B-6	20.105	25.052	0.007	−0.554	2.998
B-7	19.858	24.672	0.013	−0.514	2.971
B-8	19.939	24.977	0.027	−0.606	3.025
B-9	19.758	24.804	0.018	−0.627	3.266
B-10	19.437	24.466	0.029	−0.742	3.688
B-11	19.795	24.765	0.018	−0.636	3.224
B-12	19.489	24.449	0.013	−0.626	3.247
B-13	19.406	24.243	0.018	−0.636	3.142
B-14	19.576	24.637	0.013	−0.623	3.220
B-15	19.510	24.260	0.018	−0.622	3.095
B-16	19.394	24.206	0.022	−0.699	3.220
B-17	19.224	24.113	0.013	−0.680	3.222
B-18	19.391	24.565	0.018	−0.659	3.235
B-19	19.557	24.488	0.013	−0.674	3.168
B-20	19.723	24.743	0.019	−0.612	3.067
B-21	19.592	24.569	0.013	−0.649	3.052
B-22	19.479	24.466	0.022	−0.617	2.971
B-23	19.470	24.397	0.018	−0.683	3.072
B-24	19.095	24.044	0.022	−0.620	2.972
B-25	19.314	24.064	0.018	−0.700	3.082
B-26	19.269	24.047	0.022	−0.668	3.016
B-27	19.073	23.836	0.017	−0.662	3.084
B-28	19.154	23.752	0.021	−0.675	3.065
B-29	19.011	23.797	0.017	−0.636	3.040
B-30	19.372	24.366	0.022	−0.741	3.156
B-31	19.340	24.307	0.017	−0.652	3.060
B-32	19.225	24.374	0.024	−0.735	3.046
B-33	19.075	23.889	0.021	−0.712	3.054
B-34	19.188	23.947	0.012	−0.664	3.041
B-35	19.008	23.763	0.018	−0.631	3.073
